# Different roles of cyclic electron flow around photosystem I under sub-saturating and saturating light intensities in tobacco leaves

**DOI:** 10.3389/fpls.2015.00923

**Published:** 2015-10-27

**Authors:** Wei Huang, Ying-Jie Yang, Hong Hu, Shi-Bao Zhang

**Affiliations:** ^1^Key Laboratory of Economic Plants and Biotechnology, Kunming Institute of Botany, Chinese Academy of SciencesKunming, China; ^2^Yunnan Key Laboratory for Wild Plant ResourcesKunming, China

**Keywords:** ATP synthesis, cyclic electron flow, light acclimation, photoprotection, photorespiration, photosynthetic control

## Abstract

In higher plants, the generation of proton gradient across the thylakoid membrane (ΔpH) through cyclic electron flow (CEF) has mainly two functions: (1) to generate ATP and balance the ATP/NADPH energy budget, and (2) to protect photosystems I and II against photoinhibition. The intensity of light under which plants are grown alters both CEF activity and the ATP/NADPH demand for primary metabolic processes. However, it is unclear how the role of CEF is affected by the level of irradiance that is applied during the growth and measurement periods. We studied the role of CEF at different light intensities in leaves from sun- and shade-grown plants. At 849 μmol photons m^-2^ s^-1^, both types of leaves had nearly the same degree of CEF activation. Modeling of the ATP/NADPH demand revealed that, at this light intensity, the contribution of CEF toward supplying ATP was much higher in the sun leaves. Meanwhile, the shade leaves showed higher levels of non-photochemical quenching and the P700 oxidation ratio. Therefore, at 849 μmol photons m^-2^ s^-1^, CEF mainly helped in the synthesis of ATP in the sun leaves, but functioned in photoprotection for the shade leaves. When the light intensity increased to 1976 μmol photons m^-2^ s^-1^, CEF activation was greatly enhanced in the sun leaves, but its contribution to supplying ATP changed slightly. These results indicate that the main role of CEF is altered flexibly in response to light intensity. In particular, CEF mainly contributes to balancing the ATP/NADPH energy budget under sub-saturating light intensities. When exposed to saturating light intensities, CEF mainly protects photosynthetic apparatus against photoinhibition.

## Introduction

Light energy is harvested by photosynthetic antenna complexes. It is then used for the synthesis of ATP and NADPH, which are consumed by primary metabolic processes such as photosynthetic CO_2_ assimilation and photorespiration. However, those two processes require different ratios of ATP/NADPH that must be finely regulated, especially under strong light (Sacksteder et al., 2000; Walker et al., 2014). For example, if ATP were consumed faster than NADPH, the lack of NADP^+^ would rapidly induce the limitation of linear electron flow (LEF), decreasing proton translocation and ATP synthesis. Alternatively, if NADPH were to be consumed at a greater rate than ATP, proton translocation through ATP synthase would be reduced due to limiting ADP, This would cause an increase in the proton gradient between the thylakoid lumen and stroma (ΔpH), restricting plastoquinol oxidation at cytochrome b_6_/f and, thus, down-regulating LEF (Kanazawa and Kramer, 2002; Tikkanen and Aro, 2014). Because pool sizes of ATP and NADPH are relatively small and fluxes through primary metabolism are large (Noctor and Foyer, 2000; Avenson et al., 2005; Cruz et al., 2005; Amthor, 2010), the stoichiometric balancing of ATP and NADPH must be regulated rapidly quickly when responding to a particular level of irradiance.

In LEF, electrons are transported from water to NADP^+^, reducing the latter to NADPH. This electron transport is coupled to proton translocation and generates a ΔpH, which drives the regeneration of ATP. The stoichiometry of ATP/NADPH produced by LEF is thought to be 1.29 (Sacksteder et al., 2000; Seelert et al., 2000). However, under ambient environments, the ATP/NADPH ratio required by CO_2_ assimilation, photorespiration, and NO_3_^-^ assimilation is ∼1.6 (Edwards and Walker, 1983). If the ratio of CO_2_ to O_2_ decreases in the chloroplasts, the ATP/NADPH demand increases due to the acceleration of photorespiration (Walker et al., 2014). Such changes in energy demand require a flexible mechanism to balance ATP/NADPH. In tobacco (*Nicotiana tabacum*), plants acclimated to high light have lower concentrations of chloroplast CO_2_ (Yamori et al., 2010), leading to a higher rate of photorespiration than in plants grown under low light (Huang et al., 2014). Thus, tobacco plants exposed to stronger light utilize a flexible mechanism to balance the ATP/NADPH ratio and maintain elevated rates of CO_2_ and photorespiration.

Cyclic electron flow (CEF) around photosystem I (PSI) can balance the difference between the ATP/NADPH supply from LEF and the demand from primary metabolism (Avenson et al., 2005; Shikanai, 2007; Kramer and Evans, 2011; Walker et al., 2014). During CEF, electrons from either NADPH or ferredoxin are cycled around PSI into the plastoquinone pool, which is coupled to the generation of ΔpH but does not reducing NADP^+^ (Johnson, 2011). The role of CEF-dependent formation of ΔpH is assumed to have two main functions: (1) regeneration of ATP (Wang et al., 2006; Yamori et al., 2011; Walker et al., 2014), and (2) activation of non-photochemical quenching (NPQ) and regulation of the P700 redox state (Munekage et al., 2002, 2004; Takahashi et al., 2009; Kono et al., 2014). Therefore, CEF is considered vital for optical photosynthesis and plant growth under intense irradiation (Munekage et al., 2002, 2004; Tikkanen et al., 2014). Some studies have indicated that *PROTON GRADIENT REGULATION5* (*PGR5*)-dependent CEF has little impact on photosynthesis and LEF under high light, but can significantly affect NPQ and the P700 oxidation ratio (Takahashi et al., 2009; Nishikawa et al., 2012; Kono et al., 2014). An increase in CEF under intense light is accompanied by improved activation of NPQ (Miyake et al., 2005). Meanwhile, the rate of photorespiration is thought to be highly activated (Miyake et al., 2005), which, in turn, requires additional ATP synthesis via CEF. However, it is unclear whether the main role of CEF under high light is to balance ATP/NADPH or to activate NPQ.

The intensity of light under which plants grow has a significant effect on CEF and NPQ activities (Miyake et al., 2005). In tobacco, plants exposed to bright light have greater capacity for both CEF and NPQ when compared with plants grown under low light (Miyake et al., 2005). Furthermore, stronger irradiation enhances the capacity of the photorespiratory pathway in tobacco (Huang et al., 2014). Miyake et al. (2005) have suggested that the main role of CEF in plants acclimated to high light is to dissipate excess light energy through NPQ when illuminated at high irradiance. However, little is known about the role of CEF under sub-saturating light in plants acclimated to high light. Under low light, the rate of photosynthesis tends to be limited by the rate of ATP production rather than by the rate of NADPH production (Yamori et al., 2011). Therefore, one can assume that CEF assists with ATP synthesis under weaker light in plants acclimated to high light. By contrast, for plants acclimated to low light, the rates of photosynthesis and photorespiration are relatively low. Consequently, they should have reduced demand for CEF-dependent ATP regeneration. Thus, we speculate that, in plants exposed to low levels of light, the relative low CEF activity corresponds to the ATP demand by primary metabolisms.

The objective of the present study was to examine whether the role of CEF can be regulated flexibly under different light intensities in sun and shade leaves. We evaluated gas exchange, Chl fluorescence, and P700 parameters in leaves from tobacco plants grown under either sunny or shaded regimes. Furthermore, we used biochemical models of leaf CO_2_ assimilation to examine the rate of ATP supply from CEF. Our results indicated that the primary role of CEF is flexibly altered in response to incident light intensity.

## Materials and Methods

### Plant Materials and Growth Conditions

The seedlings of one tobacco cultivar (*Nicotiana tabacum* cv k326) were cultivated in plastic pots in a phytotron at Kunming Institute of Botany, Yunnan, China (elevation 1900 m, 102°41′E, 25°01′N). The day/night temperatures in the phytotron were 24/18°C, respectively. Relative humidity was kept at 60% and the atmospheric CO_2_ concentration (C_a_) was maintained at 400 μmol mol^-1^. The phytotron used sunlight as the source of illumination, and the intensity received by sun plants was approximately 95% of full sunlight (maximum at noon ≈ 1990 μmol photons m^-2^ s^-1^). The shade plants were grown under 28% sunlight (maximum ≈ 580 μmol photons m^-2^s^-1^). During the experimental period, none of the plants experienced any water or nutrient stresses. Full-expanded mature leaves of 14-week-old plants were used for photosynthetic measurements.

### Simultaneous Measurements of Gas Exchange and Chlorophyll Fluorescence

An open gas exchange system incorporating infrared CO_2_ and water vapor analyzers (Li-6400XT; Li-Cor Inc., Lincoln, NE, USA) was used to determine the rate of CO_2_ assimilation (*A*_n_) in the phytotron. During the measurement period, the relative humidity was 60% and air temperature was 24°C. To generate light response curves, we tested photosynthetic photon flux densities (PPFDs) of 2000, 1600, 1200, 800, 500, 300, 200, 100, 50, 20, and 0 μmol photons m^-2^ s^-1^, with a controlled *C*_a_ of 400 μmol mol^-1^. Curves for the rate of CO_2_ assimilation to the intercellular concentration of CO_2_ (*A*/*C*_i_) were measured (von Caemmerer and Farquhar, 1981) under a saturating light of 1200 μmol photons m^-2^ s^-1^. For each *A*/*C*_i_ curve, the photosynthetic rate reached a steady state at 400 μmol mol^-1^, then decreased to a low concentration of 50 μmol mol^-1^ and increased stepwise to a high concentration of 2000 μmol mol^-1^. Using those *A*/*C*_i_ curves, we calculated the maximum rates of RuBP carboxylation (*V*_cmax_) and RuBP regeneration (*J*_max_) according to the method of Long and Bernacchi (2003).

Chlorophyll fluorescence in the leaves was evaluated simultaneously with the gas exchange measurements using a leaf fluorometer chamber (6400-40; Li-Cor Inc., Lincoln, NE, USA). The fluorescence parameters *F_s_* and *F_m_′* were determined as previously described (Baker and Rosenqvist, 2004), where, after light-adaptation, *F_s_* is the steady fluorescence and *F_m_′* is the maximum fluorescence. The effective quantum yield of PSII was calculated as Φ_PSII_ = (*F_m_*′ – *F_s_*)/*F_m_*′ (Genty et al., 1989). The total photosynthetic electron flow through PSII (*J*_T_) was calculated as *J*_T_ = Φ_PSII_ × PPFD × 0.85 × 0.5 (Krall and Edwards, 1992). Because leaf absorbance (*L_abs_*) in tobacco differs little between sun and shade leaves (Miyake et al., 2005), we assumed here that *L_abs_* was 0.85 in both types. The constant of 0.5 was used based on the assumption of an equal distribution of photons between PSI and PSII (Miyake et al., 2005). On assumption that the water–water cycle in leaves is not a major alternative electron sink for dissipating excess excitation energy when CO_2_ assimilation is restricted (Driever and Baker, 2011), we allocated the electron flow through PSII to the carboxylation (*J*_C_) and oxygenation (*J*_O_) of RuBP. These were estimated according to the method of Valentini et al. (1995):

$$J_{O}=2/3\,\times \left(J_{T}-4\,\times \left(A_{net}+R_{d}\right)\right)$$

$$J_{c}=1/3\,\times \left(J_{T}-8\,\times \left(A_{net}+R_{d}\right)\right)$$

Where *A*_net_ is the measured rate of CO_2_ assimilation, *R*_d_ is the day respiration rate measured after 30 min dark adaptation.

### Modeling ATP Supplied via CEF

The ATP and NADPH demands from CO_2_ assimilation and photorespiration were calculated according to the models of Farquhar et al. (1980) and Walker et al. (2014). The total amount of electron transport required for Rubisco carboxylation and oxygenation (*J*_g_) was computed as:

$$J_{g}=\frac{\left(A_{net}\,+R_{d}\right)\,\left(4C_{i}\,+4\frac{\Gamma^{*}}{\alpha }\right)}{\left(C_{i}\,-\Gamma^{*}\right)}$$

and the NADPH demand for Rubisco carboxylation and oxygenation (ν*_NADPH_*) was determined as:

$$\nu_{NADPH}\,\;=\;0.5J_{g}$$

where C_*i*_ is the intercellular CO_2_ concentration; Γ^∗^ is the CO_2_ compensation point in the absence of day respiration, assumed to be 32.2 at 25°C (Long and Bernacchi, 2003); and α is the ratio of CO_2_ release per Rubisco oxygenation and typically assumed to be 0.5 (Farquhar et al., 1980).

The total amount of ATP demand from Rubisco carboxylation and oxygenation was obtained with the following formula:

$$\nu_{ATP}\,\;=\frac{(A_{net}\,\;+\;R_{d})\left(3C_{i}\,\;+\;3.5\frac{\Gamma^{*}}{\alpha }\right)}{\left(C_{i}\,\;-\;\Gamma^{*}\right)}$$

Finally, the rate of CEF contributing to balancing ATP and NADPH was determined by subtracting the amount of ATP produced by LEF from ν_*ATP*_ (Walker et al., 2014):

$$ATP\,\;needed\,\;from\,\;CEF\,\;=\;\nu_{ATP}\,\;-\;1.29\,\;\nu_{NADPH}$$

### Simultaneous Measurements of Chlorophyll Fluorescence and P700 Redox State

To estimate the rate of CEF around PSI, we monitored light responses in the phytotron by simultaneously obtaining chlorophyll fluorescence and the P700 redox state through the use of a Dual PAM-100 (Heinz Walz, Effeltrich, Germany). Controlled conditions included an air temperature of 24°C, relative humidity of 60%, and CO_2_ concentration of 400 μmol mol^-1^. After light adaptation at 1976 μmol photons m^-2^ s^-1^ for 20 min, the light response curves were developed for the sun- and shade-grown plants (four leaves each). The light-adapted photosynthetic parameters were assessed after 3 min of exposure to each light intensity (1976, 1618, 1311, 1052, 849, 555, 297, 150, and 77 μmol photons m^-2^ s^-1^).

Calculations were performed for two chlorophyll fluorescence parameters: Y(II), the effective quantum yield of PSII (Genty et al., 1989); and Y(NPQ), the fraction of energy that is dissipated as heat through a regulated NPQ mechanism (Hendrickson et al., 2004; Kramer et al., 2004). The following equations were used:

$$Y\left(II\right)\,=\left(F_{m}\prime -F_{s}\right)/F_{m}\prime$$

$$Y\left(NPQ\right)\,=F_{s}(F_{m}\prime -F_{s})/F_{m}$$

$$NPQ\,\;=\;\left(F_{s}\,-F_{m}\prime \right)/F_{m}\prime$$

Where, *F_m_* and *F_m_′* are the dark-adapted and light-adapted maximum fluorescence, respectively, upon illumination with a pulse (300 ms) of saturating light (10000 μmol photons m^-2^ s^-1^), and *F_s_* is the light-adapted steady-state fluorescence.

The PSI photosynthetic parameters were evaluated by Dual PAM-100, based on P700 oxidation signal (i.e., the difference in intensities of 830 and 875 nm pulse-modulated measuring light reaching the photodetector; Klüghammer and Schreiber, 2008). This useful tool for assessing the P700 redox state has been used in numerous studies (Gao et al., 2011; Yamori et al., 2011; Huang et al., 2012, 2013; Suorsa et al., 2012; Tikkanen et al., 2014). Saturation pulses (10000 μmol photons m^-2^ s^-1^), which were introduced primarily for PAM fluorescence measurements, were also applied for assessing the P700 parameters (Klüghammer and Schreiber, 1994, 2008). The P700^+^ signals (*P*) may vary between a minimum (P700 fully reduced) and a maximum level (P700 fully oxidized). The maximum, or *P_m_* (analogous to *F_m_*), is determined by applying a saturation pulse after pre-illumination with far-red light (Klüghammer and Schreiber, 2008). *P_m_*′ was determined similarly to *P_m_*, but with background actinic light instead of far-red illumination. The photochemical quantum yield of PSI, Y(I), is defined by the fraction of overall P700 that, in a given state, is reduced and not limited by the acceptor side. It is calculated as Y(I) = (*P_m_*′ -*P*)/*P_m_*. The PSI donor side limitation, Y(ND), represents the fraction of overall P700 that is oxidized in a given state. Y(ND) = *P*/*P_m_*. Both Y(I) and Y(ND) were computed automatically by the control software.

Photosynthetic electron flow through PSI and PSII were calculated as ETRI = Y(I) × PPFD × abs I × 0.5 and ETRII = Y(II) × PPFD × abs I × 0.5, where 0.5 is the proportion of absorbed light reaching PSI or PSII, and abs I is the absorbed irradiance, taken as = 0.85 of incident irradiance (Miyake et al., 2005). If CEF is functioning, ETRI will be larger than ETRII, i.e., ETRI – ETRII will show positive values. In the present study, ETRI – ETRII was used to represent the activation of CEF in sun and shade leaves.

### Statistical Analysis

All results were displayed as mean values from four to six independent experiments. The data were subjected to one-way ANOVA using SPSS 16.0 software. Statistically significant differences between sun and shade leaves were examined with *T*-tests (α = 0.05).

## Results

### CO_2_ Assimilation and Photorespiration in Sun and Shade Leaves

To calculate the electron flow devoted to RuBP oxygenation and predict ATP supplied from CEF according to CO_2_ exchange models, we measured gas exchange and chlorophyll fluorescence. In sun leaves, values were significantly higher for *R*_d_, *V*_cmax_, *J*_max_, *A*_n_, *J*_C_, *J*_O_, and *J*_O_/*J*_C_, but lower for *C*_i_ (**Tables 1** and **2**). The combination of a higher Rubisco content (as indicated by *V*_cmax_; see Yamori et al., 2010) and a lower *C*_i_ in the sun leaves caused *J*_O_ and *J*_O_/*J*_C_ to be elevated. For example, at 400 μmol mol^-1^ CO_2_ and 2000 μmol photons m^-2^ s^-1^, values for *A*_n_ were 22.2 and 12.4 μmol CO_2_ m^-2^ s^-1^ in the sun and shade leaves, respectively, whereas values for *C*_i_ were 193 (sun) and 261 μmol mol^-1^ (shade). Values for *J*_O_ were 82 and 26 μmol electrons m^-2^s^-1^ for sun and shade leaves, respectively, leading to *J*_O_/*J*_C_ ratios of 0.60 (sun) versus 0.37 (shade). The values of *J*_O_ under 800 and 2000 μmol photons m^-2^ s^-1^ differed significantly in the sun leaves. However, in the shade leaves, values of *J*_O_ under 800 and 2000 μmol photons m^-2^ s^-1^ changed slightly (**Table 2**). These results suggested that, under 2000 μmol photons m^-2^ s^-1^, the sun leaves needs more ATP supply rather than LEF to maintain the high rate of photorespiration.

**Table 1:** Values of *R*_d_, *J*_max_, and *V*_cmax_ for the sun and shade leaves.

Parameter	Sun leaves	Shade leaves
*R*_d_ (μmol CO_2_ m^-2^ s^-1^)	2.56 ± 0.23a	1.14 ± 0.19b
*J*_max_ (μmol m^-2^ s^-1^)	86.3 ± 7.4a	36.3 ± 3.8b
*V*_cmax_ (μmol m^-2^ s^-1^)	92.5 ± 8.1a	39.4 ± 3.6b


*Rates of day respiration (*R_*d*_*) were measured after dark adaptation for 30 min. Maximum rates of RuBP carboxylation (*V*_*cmax*_) and RuBP regeneration of (*J*_*max*_) were determined from photosynthetic CO_*2*_ response curves measured at 400 μmol mol^-*1*^ CO_*2*_ and 2000 μmol photons m^-*2*^ s^-*1*^. Data represent means ± SE (*n* = 4). Statistical analysis was determined between sun and shade leaves. Different characters indicate significant differences (independent *T*-test, *P* < 0.05)*

**Table 2:** Parameters from light response curves for the sun and shade leaves.

	PPFD μmol m^-2^ s^-1^	*A*_n_ μmol m^-2^ s^-1^	*C*_i_ μmol mol^-1^	*J*_C_ μmol m^-2^ s^-1^	*J*_O_ μmol m^-2^ s^-1^	*J*_O_/*J*_C_ μmol m^-2^ s^-1^	ATP from CEF μmol m^-2^ s^-1^
Sun	500	16.7 ± 0.5a	237 ± 9a	93 ± 1.5a	37 ± 1.2a	0.39 ± 0.02c	14.9 ± 0.42a
	800	19.7 ± 0.8b	212 ± 8b	116 ± 2.1b	58 ± 1.7b	0.50 ± 0.01b	18.4 ± 1.03b
	1200	21.1 ± 0.4bc	200 ± 7bc	130 ± 2.6c	75 ± 2.7c	0.57 ± 0.01a	20.3 ± 1.25bc
	2000	22.2 ± 0.6c	193 ± 8c	136 ± 7.0c	82 ± 8c	0.60 ± 0.02a	21.7 ± 1.61c
Shade	500	10.4 ± 0.8d	280 ± 8d	55 ± 4.0d	19 ± 1d	0.34 ± 0.01d	8.2 ± 1.24d
	800	11.6 ± 1.5d	270 ± 10d	63 ± 4.1e	23 ± 2.4de	0.36 ± 0.01cd	9.2 ± 1.14d
	1200	12.3 ± 1.4d	264 ± 10d	66 ± 4.3e	24 ± 2.6de	0.37 ± 0.02cd	9.9 ± 1.14d
	2000	12.4 ± 1.4d	261 ± 13d	70 ± 4.2e	26 ± 3.0e	0.37 ± 0.03cd	10.6 ± 1.27d

*Values for *A*_n_, *C*_i_, *J*_C_, *J*_O_, and *J*_O_/*J*_C_ were obtained from simultaneously measurements of gas exchange and chlorophyll fluorescence. Contribution of CEF in ATP synthesis was calculated using the method from Walker et al. (2014). Data represent means ± SE (n = 4). Statistical analysis was determined between different treatments. Different characters indicate significant differences (Tukey’s multiple comparison test, P < 0.05).*

### Modeled ATP Supplied from CEF

At 500 μmol photons m^-2^ s^-1^, the rates of ATP supplied from CEF were 14.9 and 8.2 μmol m^-2^ s^-1^ for sun and shade leaves, respectively (**Table 2**). The rates of CEF-dependent generation of ATP at 800 μmol photons m^-2^ s^-1^ were 18.4 μmol m^-2^ s^-1^ for sun leaves versus 9.2 μmol m^-2^ s^-1^ for shade leaves (**Table 2**). When exposed to 1200 μmol photons m^-2^ s^-1^, the rate of ATP from CEF in sun and shade leaves were 20.3 and 9.9 μmol m^-2^ s^-1^, respectively (**Table 2**). By comparison, upon exposure to 2000 μmol photons m^-2^ s^-1^, those rates were 21.7 μmol m^-2^ s^-1^ and 10.6 μmol m^-2^ s^-1^ in the sun and shade leaves, respectively. Thus, rates were approximately twice as high for sun leaves at both light intensities. In the sun leaves, the rate of ATP from CEF at 500 μmol photons m^-2^ s^-1^ was significantly lower than that at 800, 1200, and 2000 μmol photons m^-2^ s^-1^, and this rate changed slightly among 800, 1200, and 2000 μmol photons m^-2^ s^-1^ (**Table 2**). By contrast, the rate of ATP supplied from CEF did not differ significantly among 500, 800, 1200 and 2000 μmol photons m^-2^ s^-1^ in the shade leaves.

### Cyclic Electron Flow in Sun and Shade Leaves

**FIGURE 1:**
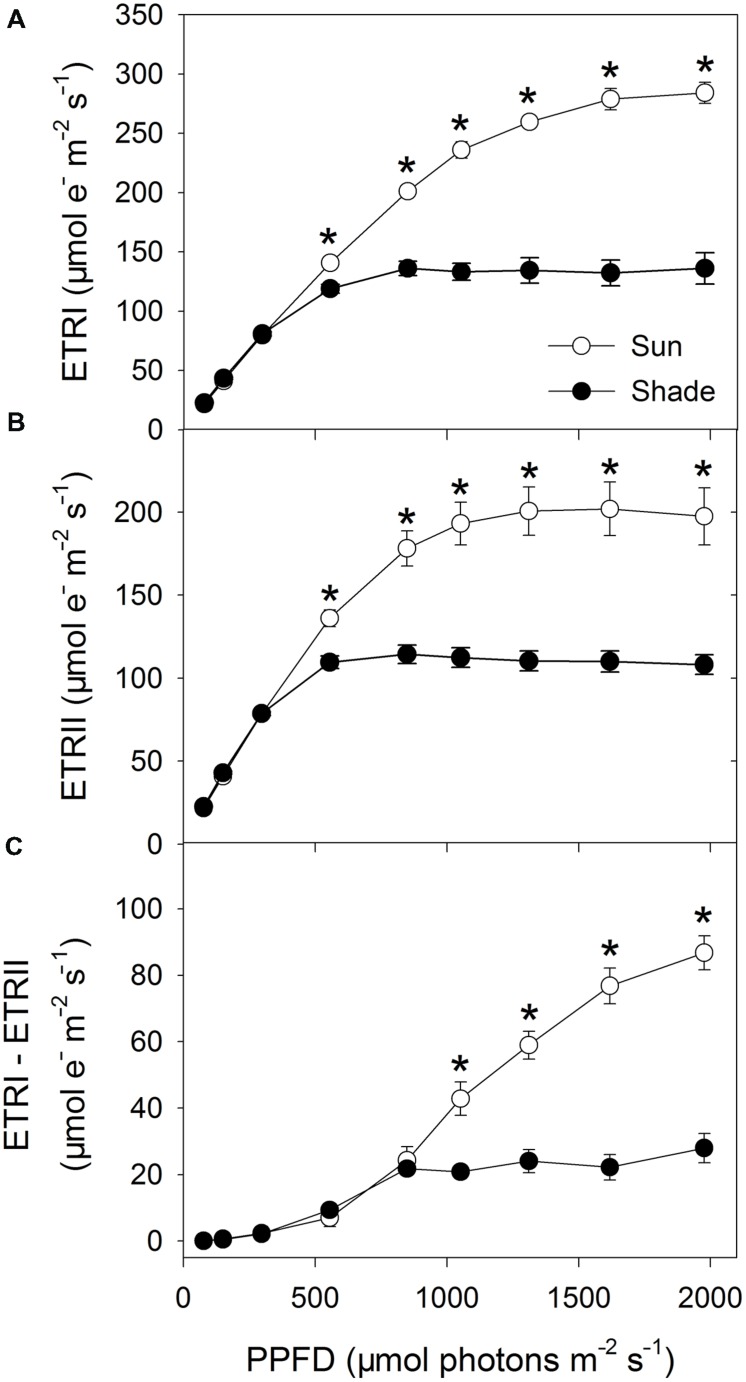
**Light response changes in ETRI **(A)**, ETRII **(B)**, and ETRI – ETRII **(C)** for leaves from sun- and shade-grown tobacco plants measured at 24°C and 400 μmol mol^-1^ CO_2_ concentration.** Values are means ± SE (*n* = 6). Significant differences between the sun and shade leaves were examined (*T*-test, *P* < 0.05). Asterisks indicate significant differences in the sun leaves compared to the shade leaves.

Values for ETRI did not differ between the sun and shade leaves under low light intensities, i.e., below 297 μmol photons m^-2^ s^-1^. However, when those intensities increased, ETRI was higher in the sun leaves (**Figure 1A**). A similar trend in light responses was found for ETRII (**Figure 1B**). CEF (as represented by ETRI – ETRII) was maintained at a low level under intensities below 555 μmol photons m^-2^ s^-1^ in both types of leaves (**Figure 1C**). At 849 μmol photons m^-2^ s^-1^, values calculated for ETRI – ETRII were similar, i.e., 24.3 μmol electrons m^-2^ s^-1^ for sun leaves and 21.7 μmol electrons m^-2^ s^-1^ for shade leaves. Under stronger light, however, the rise in CEF (ETRI – ETRII) was large for sun leaves but only slight for shade leaves. For example, at 1976 μmol photons m^-2^ s^-1^, the CEF was 86.8 μmol electrons m^-2^ s^-1^ for sun leaves and only 28.0 μmol electrons m^-2^ s^-1^ for shade leaves.

### Non-photochemical Quenching and P700 Oxidation Ratio

**FIGURE 2:**
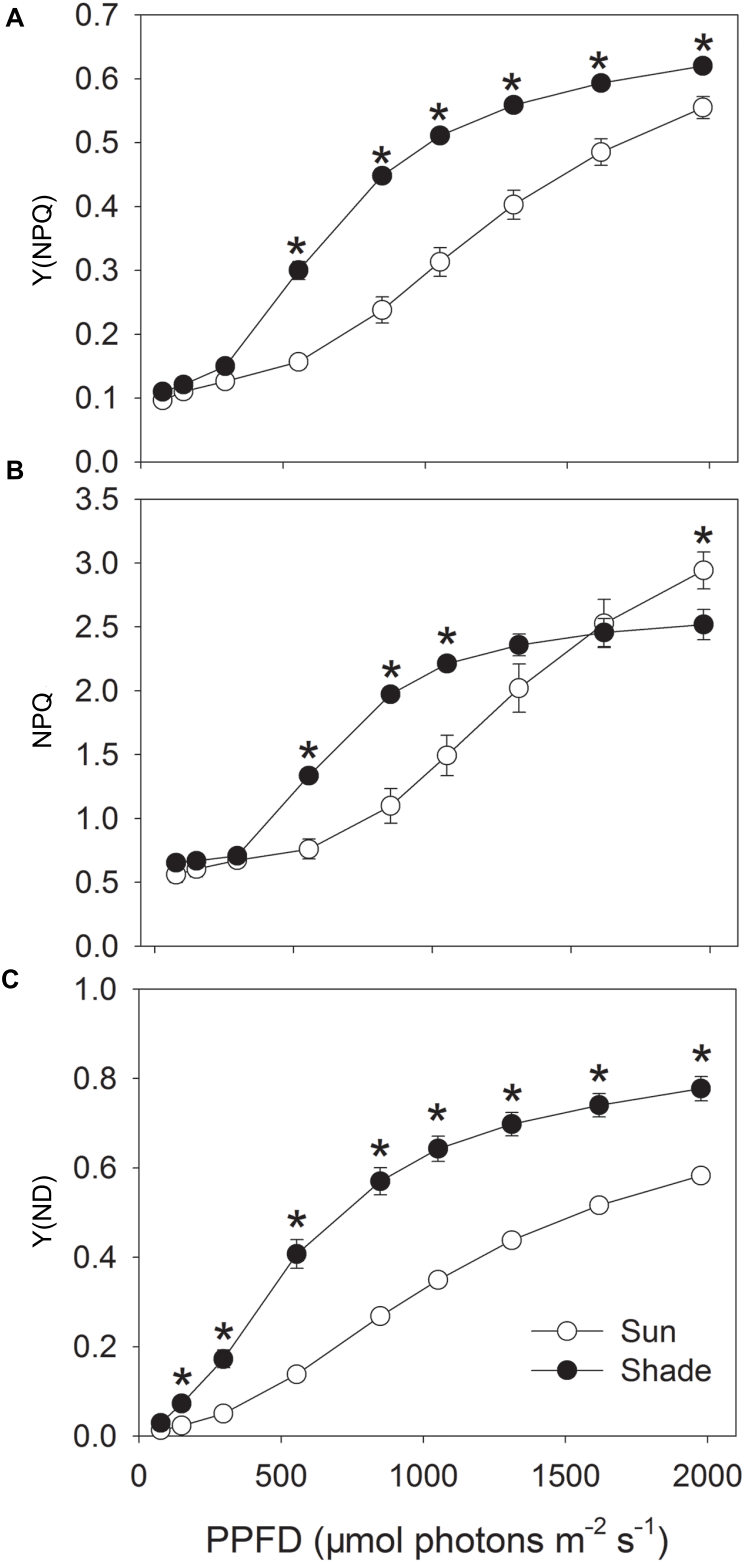
**Light response changes in Y(NPQ) **(A)**, NPQ **(B)** and Y(ND) **(C)** for leaves from sun- and shade-grown tobacco plants measured at 24°C and 400 μmol mol^-1^ CO_2_ concentration.** Values are means ± SE (*n* = 6). Values are means ± SE (*n* = 6). Significant differences between the sun and shade leaves were examined (*T*-test, *P* < 0.05). Asterisks indicate significant differences in the sun leaves compared to the shade leaves.

Non-photochemical quenching can be subdivided into three components: energy-dependent thermal dissipation (qE), photoinhibition (qI), and state transitions (qT). Under saturating light conditions, qE is the major component of NPQ (Takahashi et al., 2009; Kono et al., 2014). Activation of qE is regulated by low lumenal pH, which is accompanied by the generation of ΔpH. As a result, the absolute value of NPQ can reflect the difference in ΔpH (Kono et al., 2014). For both sun and shade leaves, Y(NPQ) was maintained at a low level under light intensities less than 297 μmol photons m^-2^ s^-1^ (**Figure 2A**). At 555 μmol photons m^-2^ s^-1^, the fraction of energy dissipated as heat via the regulated NPQ mechanism [Y(NPQ)] was greatly increased in the shade leaves but remained constant in the sun leaves (0.30 for shade versus 0.16 for sun). In both sun and shade leaves exposed to light intensities above 555 μmol photons m^-2^ s^-1^, Y(NPQ) gradually rose, but was significantly higher in the shade leaves (**Figure 2A**). Values for NPQ were maintained at low levels in both type leaves when exposed to low light intensities below 297 μmol photons m^-2^ s^-1^ (**Figure 2B**), suggesting that the sun and shade leaves had similar values for qI and qT since qI and qT change little under different light intensities (Kono et al., 2014). Similar to the trend of Y(NPQ), the shade leaves had higher NPQ than the sun leaves at light intensities of 555, 849, 1052, 1311 μmol photons m^-2^ s^-1^ (**Figure 2B**), suggesting the higher ΔpH in the shade leaves at these light intensities. At 1976 μmol photons m^-2^ s^-1^, the sun leaves had significantly higher NPQ than the shade leaves, which was different from the trend of Y(NPQ). With increase in light intensity, NPQ gradually increased in the sun leaves and did not reach the maximum value at 1976 μmol photons m^-2^ s^-1^. On the contrary, NPQ nearly reached its maximum value at 1311 μmol photons m^-2^ s^-1^ in the shade leaves (**Figure 2B**).

The fraction of overall P700 that is oxidized in a given state [Y(ND)] followed a trend similar to that for Y(NPQ) (**Figure 2C**), remaining at a low level in both leaf types under weak irradiance. As the light became more intense, Y(ND) also gradually increased. Values were much higher in the shade leaves than in the sun leaves, especially under high light. These results indicated that the shade leaves up-regulated NPQ and the P700 oxidation ratio under high light to prevent over-reduction of the electron transfer chain.

**FIGURE 3:**
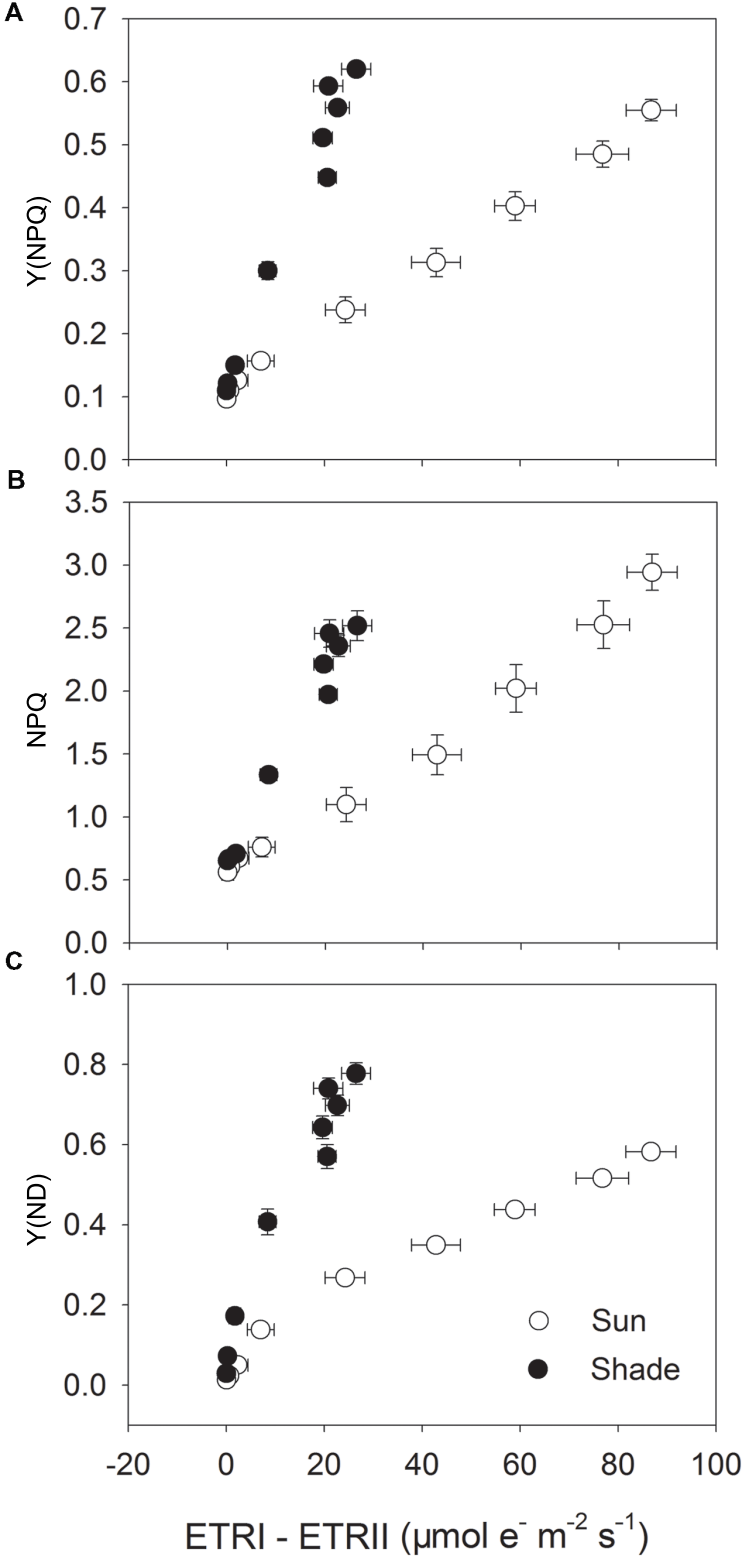
**Relationship between ETRI – ETRII and Y(NPQ) **(A)**, NPQ **(B)** and Y(ND) **(C)** for sun and shade leaves.** Light response data in **Figures 1** and **2** were used.

To examine the role of CEF-dependent generation of ΔpH in the regulation of photosynthetic electron flow, we investigated the relationships between ETRI – ETRII and Y(NPQ), NPQ or Y(ND) (**Figure 3**). Activation of Y(NPQ) and NPQ was positively and significantly correlated with that of CEF. However, under strong light, the same value of ETRI – ETRII was accompanied by a higher Y(NPQ) and NPQ in the shade leaves (**Figures 3A,B**). This relationship was similar between ETRI – ETRII and Y(ND) (**Figure 3C**). Because the high values for Y(NPQ), NPQ, and Y(ND) were both largely dependent on CEF-dependent generation of ΔpH (Munekage et al., 2002, 2004; Kono et al., 2014), the CEF-dependent generation of ΔpH mainly functioned in photoprotection in the shade leaves through activating NPQ and increasing P700 oxidation ratio. When compared with the sun leaves, the contribution of CEF to ATP synthesis was small in the shade leaves.

## Discussion

The contrast in values for *J*_O_/*J*_C_ between sun and shade leaves appears to be linked to different ATP/NADPH demands. Furthermore, their different capacities to utilize light energy mean that sun and shade leaves do not require the same level of photoprotection at a given light. Although CEF activation is involved in ATP synthesis and photoprotective functions, the specific role of CEF under different light intensities in each leaf type is unclear. This work was to test whether the role of CEF can be regulated flexibly in response to incident light in the sun and shade leaves. Our results indicate that, CEF primarily assists in maintaining a balance in the ATP/NADPH ratio under sub-saturating light conditions but tends to mainly participate in photoprotection for PSI and PSII under saturating light conditions.

In both leaf types, CEF (as represented by ETRI – ETRII) was low at light intensities less than 555 μmol photons m^-2^ s^-1^ but was significantly activated when plants were exposed to light intensities above 849 μmol photons m^-2^ s^-1^. At 849 μmol photons m^-2^ s^-1^, similar values for CEF were obtained in sun and shade leaves. Under stronger light, however, the rise in CEF was very large for sun leaves but only slight for shade leaves. Our results are consistent with those calculated for wild-type plants of *Arabidopsis thaliana*, in which the ETRI/ETRII ratio is close to 1.0 under weaker light (<100 μmol photons m^-2^ s^-1^) but elevated as PPFD increases (Kono et al., 2014). Furthermore, these results support an earlier conclusion by Miyake et al. (2005) that CEF activity is obviously greater in sun leaves than in shade leaves of tobacco. Therefore, our estimates of CEF in sun and shade leaves are reliable.

Comparing with shade leaves, the higher Rubisco content and lower *C*_i_ in the sun leaves induced higher rate of photorespiration (**Table 2**), which requires a larger ATP/NADPH ratio to maintain high levels of CO_2_ assimilation, photorespiration, and photosynthetic electron flow (Edwards and Walker, 1983; Walker et al., 2014). However, the stoichiometry of the ATP/NADPH ratio produced by LEF is thought to be 1.29. Such a change in energy demand necessitates a flexible mechanism to add ATP synthesis and then balance that ratio. In addition to LEF, the CEF-dependent generation of ΔpH can support ATP synthesis (Shikanai, 2007; Kramer and Evans, 2011; Yamori et al., 2011; Wang et al., 2015). We found that, at 849 μmol photons m^-2^ s^-1^, CEF activation was similar between sun and shade leaves, but twice as much ATP was supplied from CEF in the sun leaves. These findings demonstrated that CEF-dependent generation of ΔpH mainly contributed to ATP synthesis under that level of irradiance in the sun leaves. The supplemental ATP synthesis induced by CEF was used to balance the difference between ATP/NADPH supply from LEF and the demand from CO_2_ assimilation and photorespiration in the sun leaves. If this did not occur, then the electron transfer from H_2_O to NADP^+^ would have been rapidly suppressed due to a lack of NADP^+^, thereby restricting CO_2_ assimilation and photorespiration. Because the Rubisco content was lower in the shade leaves, the rate of CO_2_ assimilation was restricted (**Table 2** and Yamori et al., 2010), leading to a higher *C*_i_ and, thus, a reduced rate of photorespiration (**Table 2** and Huang et al., 2014). Consequently, the stoichiometry of the ATP/NADPH ratio required for primary metabolism was relatively lower in the shade leaves. Accordingly, the rate of ATP supplied from CEF was relatively lower.

We found it interesting that, in the sun leaves, the rate of ATP supplied from CEF differed only slightly between 800, 1200 and 2000 μmol photons m^-2^ s^-1^ but CEF activation was markedly changed between 849, 1311 and 1976 μmol photons m^-2^ s^-1^. These results suggested that, in the sun leaves, CEF-dependent generation of ΔpH mainly helps in providing ATP under sub-saturating light conditions such as 800 μmol photons m^-2^ s^-1^, thereby balancing the ratio of ATP/NADPH needed for CO_2_ assimilation and photorespiration. Under saturating photosynthetic electron fluxes such as 2000 μmol photons m^-2^ s^-1^, CEF-dependent generation of ΔpH is primarily involved in photoprotection for PSI and PSII. This hypothesis is supported by research with mutants that lack key enzymes for the CEF pathway. For example, the PGR5 mutant (*pgr5*) from *Arabidopsis thaliana* is viable under low light but displays severe photoinhibition of PSI and PSII under high light (Munekage et al., 2002; Takahashi et al., 2009). When compared with the wild type, this mutant has a reduced rate of photosynthesis at irradiances below 370 μmol photons m^-2^ s^-1^, but a similar rate of CO_2_ assimilation under 1000 μmol photons m^-2^ s^-1^ (Nishikawa et al., 2012). Photosynthesis in a rice mutant lacking the NADPH-dependent pathway of CEF (*ccr6*) is reduced under light intensities of less than 500 μmol photons m^-2^ s^-1^, but its photosynthetic rate is similar to that of the wild type under stronger irradiance (>1000 μmol photons m^-2^ s^-1^; Yamori et al., 2011).

For tobacco plants, light saturating point (LSP) of CO_2_ assimilation is lower in the shade leaves than the sun leaves (Huang et al., 2014). For example, 849 μmol photons m^-2^ s^-1^ is higher than LSP of the shade leaves but lower than LSP of the sun leaves. In the shade-grown tobacco leaves, a low level of CEF activation under low light intensities below 555 μmol photons m^-2^ s^-1^ may be involved in ATP synthesis, accompanying with low levels of Y(NPQ) and Y(ND). Once CEF was largely activated under saturating light such as 849 μmol photons m^-2^ s^-1^, Y(NPQ) and Y(ND) largely increased. At 849 μmol photons m^-2^ s^-1^, CEF activation was similar between the sun and shade leaves, but Y(NPQ) and Y(ND) were significantly higher in the shade leaves. This indicated that, when exposed to saturating light intensity such as 849 μmol photons m^-2^ s^-1^, the main role of CEF in the shade leaves was to function for photoprotection.

Under saturating light, CO_2_ assimilation is limited by RuBP regeneration and/or RuBP carboxylation. Interruption of the Calvin cycle inhibits the repair of photodamaged PSII (Takahashi and Murata, 2005). To diminish the production of reactive oxygen species (ROS), leaves had to activate NPQ strongly. Activation of NPQ is based on the generation of ΔpH through LEF and CEF (Munekage et al., 2002, 2004; Takahashi et al., 2009; Suorsa et al., 2012). The activation of CEF can increase the ΔpH, which causes the major light chlorophyll a/b light harvesting antenna protein (LCHII) to dissipate excess excitation energy harmlessly as heat. Subsequently, the energy transfer efficiency from LCHII to the photosystems is reduced (Tikkanen and Aro, 2014), which then decreases ROS production and favors the repair of PSII. Furthermore, the CEF-dependent generation of ΔpH can limit the extent of photodamage to PSII by protecting the oxygen-evolving complex (Takahashi et al., 2009). Because PSI tends to be damaged when electron flow from PSII to PSI exceeds the capacity of PSI electron acceptors (Tikkanen et al., 2014), the rise in ΔpH due to CEF activation under saturating light conditions can control the electron flow from PSII to PSI via the Cyt b_6_/f complex, thereby protecting PSI against photodamage (Suorsa et al., 2012, 2013; Tikkanen and Aro, 2014; Tikkanen et al., 2015). Furthermore, because over-reduction on the PSI acceptor side can lead to PSI photodamage (Munekage et al., 2002; Suorsa et al., 2012; Kono et al., 2014; Tikkanen et al., 2014), CEF activation under strong light can prevent over-reduction on the PSI acceptor side, as indicated by the high P700 oxidation ratio calculated here. In the CEF (*pgr5*) mutant of *Arabidopsis thaliana*, Y(NPQ) and Y(ND) are maintained at low levels under high light (Kono et al., 2014), leading to severe photodamage to PSI (Munekage et al., 2002; Suorsa et al., 2012) and PSII (Takahashi et al., 2009). Thus, we conclude that the main role of CEF under saturating light is to protect PSI and PSII against photodamage.

## Conclusion

Our results strongly suggest that the main function of CEF is flexibly changed according to lighting conditions in sun and shade leaves of tobacco. Under sub-saturating light intensities, CEF-dependent generation of ΔpH mainly contributes to ATP synthesis and regulates ATP/NADPH ratio, which optimize photosynthetic CO_2_ assimilation and photosynthetic electron flow. Under saturating light intensities, the ability of leaves to utilize light energy is restricted by RuBP carboxylation and/or RuBP regeneration. Meanwhile, CEF-dependent generation of ΔpH primarily activates NPQ, regulates P700 redox state and controls the transfer of electrons via the Cyt b_6_f complex, which then protects PSI and PSII against photodamage. Enhanced CEF activity in the sun leaves is an important mechanism that alternates between balancing ATP/NADPH and functioning in photoprotection under different light scenarios.

## Conflict of Interest Statement

The authors declare that the research was conducted in the absence of any commercial or financial relationships that could be construed as a potential conflict of interest.

## References

[B1] Amthor J. S. (2010). From sunlight to phytomass: on the potential efficiency of converting solar radiation to phyto-energy. *New Phytol.* 188 939–959. 10.1111/j.1469-8137.2010.03505.x 20977480

[B2] Avenson T. J. Cruz J. A. Kanazawa A. Kramer D. M. (2005). Regulating the proton budget of higher plant photosynthesis. *Proc. Natl. Acad. Sci. U.S.A.* 102 9709–9713. 10.1073/pnas.0503952102 15972806 PMC1172270

[B3] Baker N. R. Rosenqvist E. (2004). Applications of chlorophyll fluorescence can improve crop production strategies: an examination of future possibilities. *J. Exp. Bot.* 55 1607–1621. 10.1093/jxb/erh196 15258166

[B4] Cruz J. A. Avenson T. J. Kanazawa A. Takizawa K. Edwards G. E. Kramer D. M. (2005). Plasticity in light reactions of photosynthesis for energy production and photoprotection. *J. Exp. Bot.* 56 395–406. 10.1093/jxb/eri022 15533877

[B5] Driever S. M. Baker N. R. (2011). The water-water cycle in leaves is not a major alternative electron sink for dissipation of excess excitation energy when CO2 assimilation is restricted. *Plant Cell Environ.* 34 837–846. 10.1111/j.1365-3040.2011.02288.x 21332508

[B6] Edwards G. E. Walker D. A. (1983). *C3 C4: Mechanisms, and Cellular and Environmental Regulation, of Photosynthesis*. Oxford: Blackwell Scientific.

[B7] Farquhar G. D. von Caemmerer S. Berry J. A. (1980). A biochemical model of photosynthetic CO2 assimilation in leaves of C3 species. *Planta* 149 78–90. 10.1007/BF00386231 24306196

[B8] Gao S. Shen S. D. Wang G. C. Niu J. F. Lin A. P. Pan G. H. (2011). PSI-driven cyclic electron flow allows intertidal macro-algae Ulva sp. (Chlorophyta) to survive in desiccated conditions. *Plant Cell Physiol.* 52 885–893. 10.1093/pcp/pcr038 21471121

[B9] Genty B. Briantais J. M. Baker N. R. (1989). The relationship between the quantum yield of photosynthetic electron transport and quenching of chlorophyll fluorescence. *Biochim. Biophys. Acta* 99 87–92. 10.1016/S0304-4165(89)80016-9

[B10] Hendrickson L. Furbank R. T. Chow W. S. (2004). A simple alternative approach to assessing the fate of absorbed light energy using chlorophyll fluorescence. *Photosynth. Res.* 82 73–81. 10.1023/B:PRES.0000040446.87305.f4 16228614

[B11] Huang W. Fu P. L. Jiang Y. J. Zhang J. L. Zhang S. B. Hu H. (2013). Differences in the responses of photosystem I and photosystem II of three tree species *Cleistanthus sumatranus*, *Celtis philippensis* and *Pistacia weinmannifolia* submitted to a prolonged drought in a tropical limestone forest. *Tree Physiol.* 33 211–220. 10.1093/treephys/tps132 23329334

[B12] Huang W. Yang S. J. Zhang S. B. Zhang J. L. Cao K. F. (2012). Cyclic electron flow plays an important role in photoprotection for the resurrection plant *Paraboea rufescens* under drought stress. *Planta* 235 819–828. 10.1007/s00425-011-1544-3 22080919

[B13] Huang W. Zhang S. B. Hu H. (2014). Sun leaves up-regulate the photorespiratory pathway to maintain a high rate of CO2 assimilation in tobacco. *Front. Plant Sci.* 5:688 10.3389/fpls.2014.00688 PMC4253947 25520735

[B14] Johnson G. N. (2011). Physiology of PSI cyclic electron transport in higher plants. *Biochim. Biophys. Acta* 1807 384–389. 10.1016/j.bbabio.2010.11.009 21118673

[B15] Kanazawa A. Kramer D. M. (2002). In vivo modulation of nonphotochemical exciton quenching (NPQ) by regulation of the chloroplast ATP synthase. *Proc. Natl. Acad. Sci. U.S.A.* 99 12789–12794. 10.1073/pnas.182427499 12192092 PMC130538

[B16] Klüghammer C. Schreiber U. (1994). An improved method, using saturating light pulses, for the determination of photosystem I quantum yield via P700+-absorbance changes at 830 nm. *Planta* 192 261–268. 10.1007/BF01089043

[B17] Klüghammer C. Schreiber U. (2008). Saturation pulse method for assessment of energy conversion in PSI. *PAM Appl. Notes* 1 11–14.

[B18] Kono M. Noguchi K. Terashima I. (2014). Roles of the cyclic electron flow around PSI (CEF-PSI) and O2-dependent alternative pathways in regulation of the photosynthetic electron flow in short-term fluctuating light in *Arabidopsis* thaliana. *Plant Cell Physiol.* 55 990–1004. 10.1093/pcp/pcu033 24553846

[B19] Krall J. P. Edwards G. E. (1992). Relationship between photosystem II activity and CO2 fixation in leaves. *Physiol. Plant.* 86 180–187. 10.1007/BF00016557

[B20] Kramer D. M. Evans J. R. (2011). The importance of energy balance in improving photosynthetic productivity. *Plant Physiol.* 155 70–78. 10.1104/pp.110.166652 21078862 PMC3075755

[B21] Kramer D. M. Johnson G. Kiirats O. Edwards G. E. (2004). New fluorescence parameters for the determination of QA redox state and excitation energy fluxes. *Photosynth. Res.* 79 209–218. 10.1023/B:PRES.0000015391.99477.0d 16228395

[B22] Long S. P. Bernacchi C. J. (2003). Gas exchange measurements, what can they tell us about the underlying limitations to photosynthesis? Procedures and sources of error. *J. Exp. Bot.* 54 2393–2401. 10.1093/jxb/erg262 14512377

[B23] Miyake C. Miyata M. Shinzaki Y. Tomizawa K. (2005). CO2 response of cyclic electron flow around PSI (CEF-PSI) in tobacco leaves—relative electron fluxes through PSI and PSII determine the magnitude of non-photochemical quenching (NPQ) of chl fluorescence. *Plant Cell Physiol.* 46 629–637. 10.1093/pcp/pci197 15701657

[B24] Munekage Y. Hashimoto M. Miyake C. Tomizawa K. I. Endo T. Tasaka M. (2004). Cyclic electron flow around photosystem I is essential for photosynthesis. *Nature* 429 579–582. 10.1038/nature02598 15175756

[B25] Munekage Y. Hojo M. Meurer J. Endo T. Tasaka M. Shikanai T. (2002). PGR5 is involved in cyclic electron flow around photosystem I and is essential for photoprotection in *Arabidopsis*. *Cell* 110 361–371. 10.1016/S0092-8674(02)00867-X 12176323

[B26] Nishikawa Y. Yamamoto H. Okegawa Y. Wada S. Sato N. Taira Y. (2012). PGR5-dependent cyclic electron transport around PSI contributes to the redox homeostasis in chloroplasts rather than CO2 fixation and biomass production in rice. *Plant Cell Physiol.* 53 2117–2126. 10.1093/pcp/pcs153 23161858

[B27] Noctor G. Foyer C. H. (2000). Homeostasis of adenylate status during photosynthesis in a fluctuating environment. *J. Exp. Bot.* 51 347–356. 10.1093/jexbot/51.suppl_1.347 10938842

[B28] Sacksteder C. A. Kanazawa A. Jacoby M. E. Kramer D. M. (2000). The proton to electron stoichiometry of steady-state photosynthesis in living plants: a proton-pumping Q cycle is continuously engaged. *Proc. Natl. Acad. Sci. U.S.A.* 97 14283–14288. 10.1073/pnas.97.26.14283 11121034 PMC18910

[B29] Seelert H. Poetsch A. Dencher N. A. Engel A. Stahlberg H. Müller D. J. (2000). Proton-powered turbine of a plant motor. *Nature* 405 418–419. 10.1038/35013148 10839529

[B30] Shikanai T. (2007). Cyclic electron transport around photosystem I: genetic approaches. *Annu. Rev. Plant. Biol.* 58 199–217. 10.1146/annurev.arplant.58.091406.110525 17201689

[B31] Suorsa M. Grieco M. Jarvi S. Gollan P. J. Kangasjarvi S. Tikkanen M. (2013). PGR5 ensures photosynthetic control to safeguard photosystem I under fluctuating light conditions. *Plant. Signal. Behav.* 8 e22741. 10.4161/psb.22741 PMC3745580 23221748

[B32] Suorsa M. Jarvi S. Grieco M. Nurmi M. Pietrzykowska M. Rantala M. (2012). PROTON GRADIENT REGULATION5 is essential for proper acclimation of *Arabidopsis* photosystem I to naturally and artificially fluctuating light conditions. *Plant Cell* 24 2934–2948. 10.1105/tpc.112.097162 22822205 PMC3426124

[B33] Takahashi S. Milward S. E. Fan D. Y. Chow W. S. Badger M. R. (2009). How does cyclic electron flow alleviate photoinhibition in *Arabidopsis*? *Plant Physiol.* 149 1560–1567. 10.1104/pp.108.134122 19118124 PMC2649389

[B34] Takahashi S. Murata N. (2005). Interruption of the Calvin cycle inhibits the repair of photosystem II from photodamage. *Biochim. Biophys. Acta* 1708 352–361. 10.1016/j.bbabio.2005.04.003 15955527

[B35] Tikkanen M. Aro E. M. (2014). Integrative regulatory network of plant thylakoid energy transduction. *Trends Plant Sci.* 19 10–17. 10.1016/j.tplants.2013.09.003 24120261

[B36] Tikkanen M. Mekala N. R. Aro E. M. (2014). Photosystem II photoinhibition-repair cycle protects Photosystem I from irreversible damage. *Biochim. Biophys. Acta* 1837 210–215. 10.1016/j.bbabio.2013.10.001 24161359

[B37] Tikkanen M. Rantala S. Aro E. M. (2015). Electron flow from PSII to PSI under high light is controlled by PGR5 but not by PSBS. *Front. Plant Sci.* 6:521 10.3389/fpls.2015.00521 PMC4495676 26217370

[B38] Valentini R. Epron D. De Angelis P. Matteucci G. Dreyer E. (1995). In situ estimation of net CO2 assimilation, photosynthetic electron flow and photorespiration in Turkey oak (Q. cerris L.) leaves: diurnal cycles under different levels of water supply. *Plant Cell Environ.* 18 631–640. 10.1111/j.1365-3040.1995.tb00564.x

[B39] von Caemmerer S. Farquhar G. D. (1981). Some relationships between the biochemistry of photosynthesis and the gas exchange of leaves. *Planta* 153 376–387. 10.1007/BF00384257 24276943

[B40] Walker B. J. Strand D. D. Kramer D. M. Cousins A. B. (2014). The response of cyclic electron flow around photosystem I to changes in photorespiration and nitrate assimilation. *Plant Physiol.* 165 453–462. 10.1104/pp.114.238238 24664207 PMC4012602

[B41] Wang C. Yamamoto H. Shikanai T. (2015). Role of cyclic electron transport around photosystem I in regulating proton motive force. *Biochim. Biophys. Acta* 1847 931–938. 10.1016/j.bbabio.2014.11.013 25481109

[B42] Wang P. Duan W. Takabayashi A. Endo T. Shikanai T. Ye J. Y. (2006). Chloroplastic NAD(P)H dehydrogenase in tobacco leaves functions in alleviation of oxidative damage caused by temperature stress. *Plant Physiol.* 141 465–474. 10.1104/pp.105.070490 16428601 PMC1475475

[B43] Yamori W. Evans J. R. von Caemmerer S. (2010). Effects of growth and measurement light intensities on temperature dependence of CO2 assimilation rate in tobacco leaves. *Plant Cell Environ.* 33 332–343. 10.1111/j.1365-3040.2009.02067.x 19895395

[B44] Yamori W. Sakata N. Suzuki Y. Shikanai T. Makino A. (2011). Cyclic electron flow around photosystem I via chloroplast NAD(P)H dehydrogenase (NDH) complex performs a significant physiological role during photosynthesis and plant growth at low temperature in rice. *Plant J.* 68 966–976. 10.1111/j.1365-313X.2011.04747.x 21848656

